# Freshwater crabs could act as vehicles of spreading avian influenza virus

**DOI:** 10.1186/s12985-021-01708-8

**Published:** 2021-12-11

**Authors:** Weiwei Ma, Chenyang Ren, Qingbiao Hu, Xiaodong Li, Yali Feng, Ying Zhang

**Affiliations:** Key Laboratory of Livestock Infectious Diseases, Ministry of Education, Key Laboratory of Zoonosis, College of Animal Science and Veterinary Medicine, Liaoning Panjin Wetland Ecosystem National Observation and Research Station, Shenyang Agricultural University, 120 Dongling Rd, Shenyang, 110866 People’s Republic of China

**Keywords:** Avian influenza virus, Freshwater crab, Aquatic, Spread

## Abstract

Avian influenza virus (AIV) possessed significant risk to various animals and human health. Wild birds, especially waterfowls are considered to be the natural reservoir of AIVs. The ecology of AIV is still far from being fully understood. Freshwater crabs are nonnegligible biotic factor in AIV ecosystem. We analyzed the ability of freshwater crabs accumulate and spread AIV. We found that AIV remain infectious in water only for 36 h but persist in crabs for 48 h. Crabs could accumulate AIV in their gills and gastrointestinal tracts. The AIV titers in crabs were higher than the surrounding contaminated water. Crabs could accumulate AIV from contaminated water, carry the virus and spread to naïve crabs via surrounding water. Our study identified freshwater crab as a novel transmission vehicle in AIV ecosystem.

## Introduction

Avian Influenza virus (AIV) is important pathogen for both human and animals. AIV is a RNA virus composed of 8 genome segments. Hemagglutinin (HA) and neuraminidase (NA) are two major glycoproteins on the surface of AIV. AIV can be classified into 18 HA (H1-H18) and 11 NA (N1-N11) subtypes based on the different antigenicity of HA and NA. All of these subtypes have been found in wild birds excepting for H17N10 and H18N11 subtypes [1, 2]. So it has been widely accepted that wild birds, especially waterfowls are the natural reservoir of AIVs. Occasionally, AIV in waterfowls could spill over to domestic poultry, livestock, marine mammals and even humans [3, 4].

AIVs mainly replicate in the intestine tract cells of waterfowls [5], so the infected bird faeces may contain high concentration of AIVs. The faecal-oral route is considered to be the primary AIV transmission mode in waterfowls. It has been reported that AIVs can remain infectious in aquatic environment for more than seven months [6]. As a result, it makes the aquatic environment become an epidemic focus where AIV could transmit among waterfowl and other animal living in the same area [7].

Freshwater crabs are widely existing in freshwater lakes, rivers and brackish waters [8]. It is an omnivorous animal which mainly feed on plant and animal detritus [9]. In the wild, the crabs could also be the prey of waterfowl and poultry. Sharing same aquatic habitats and being in predation relation with waterfowls, freshwater crabs might become a transmission biotic factor in AIV ecosystem. Chinese mitten crab (*Eriocheir sinensis*) is the main freshwater crab species in China. It belongs to *Malacostraca*, *Decapoda*, *Grapsidae*. In this study we used Chinese mitten crabs as a representative model to evaluate the function of freshwater crab in AIV ecosystem.

## Materials and methods

### Freshwater crabs

The Chinese mitten crabs used in this experiment weighed 10.0 ± 1.0 g were kindly provided by Panjin Guanghe Crab industry Co Ltd. The crabs were kept in aerated water for 2 weeks at 18 °C.

Aerated water was made by pumping air into 20 Liter tap water with an air pump (20 L/min) overnight. Finally, the dissolved oxygen and pH level of the aerated water was 6.4 mg/mL and 7.2 respectively.

### Virus and cell

H9N2 avian influenza virus A/chicken/Liaoning/07/2016 was isolated from chicken during routine surveillance. Virus stock was propagated in Madin-Darby canine kidney (MDCK) cells and stored at -80 °C. The viral titer was determined by 50% tissue culture infectious dose (TCID_50_).

### Persistence of Avian influenza virus in the aerated water

The viral water was made by adding 10^7.5^ TCID_50_ of AIV into 1 Liter aerated water, mixing thoroughly. Three tanks with 1 L viral water were put into a biosafety cabinet at 18 °C. 1 ml water sample was taken from each tank after 0, 1, 3, 8, 12, 24, 36, 48 and 60 hours (h) respectively to test the viral titer in MDCK cells.

### AIV accumulation in freshwater crabs

Five groups crabs (3 per group) were distributed into five viral water tanks. At 0, 1, 3, 8 and 12 h post incubation (hpi), one group of three crabs were rinsed and euthanized. Crabs gills, hepatopanpancreas, gastrointestinals, muscles and viral water sample were collected for viral titration.

### AIV accumulating limitation of crabs

Six groups of crabs (3 per group) were incubated in viral water. Every 12 h, the tank water was changed with fresh viral water until 60 h later. Since 0 h after incubation, one group of crabs were rinsed and euthanized for gills collection before water changing. The viral water sample was collected at the same time.

### AIV spreading activity of freshwater crabs

Groups of 3 crabs were incubated in viral water for 8 h as inoculated groups. After rinsing thoroughly, the inoculated crabs were transferred into fresh water. Groups of 3 naïve crabs, as sentinel groups, were put into each tank and co-cultured with inoculated groups. The inoculated and sentinel groups crabs’ samples were collected immediately after co-cultured. During the first 4 h post co-cultured (hpc), every 0.5 h 1 group of inoculated and sentinel crabs were rinsed and euthanized. Their gills and water sample were collected at the same time for viral titration. The crabs and water samples were also collected at 8, 12, 24, 36, 48 and 60 hpc respectively.

## Results

### Infectivity changes of AIV in lab water tank over time

**Fig. 1:**
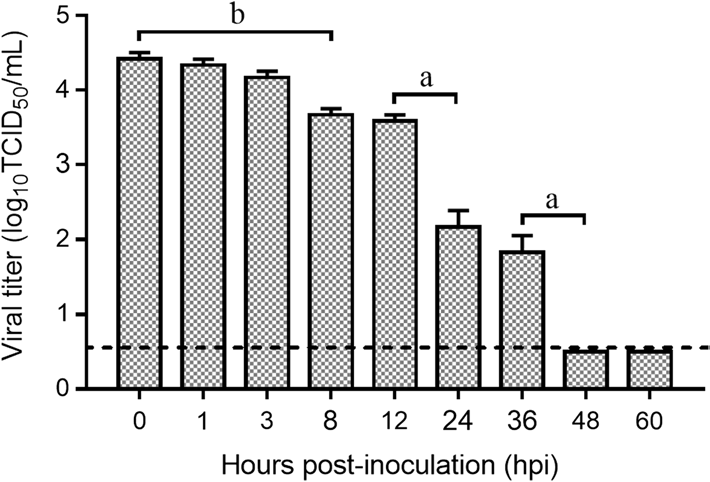
Persistence of AIV in aerated water. 10^7.5^TCID_50_ AIV virus was added to 1 L aerated water at 18 °C. Water samples were collected at designated time points and titrated on MDCK cells. The data shown are the means of three replicates; the error bars indicate standard deviations. The dashed lines indicate the lower limit of virus detection. Data were analyzed using analysis of variance (ANOVA) in GraphPad Prism version 9.0 (GraphPad Software Inc., CA, USA). Significance was analyzed by using a one-way ANOVA with post-hoc tests. a, P < 0.001; b, P < 0.05

As shown in Fig. 1, AIV could maintain similar infectivity for 3 h. Viral titer began to drop at 8 hpi. At 24 hpi, viral titer dropped by half. There’s no detectable virus in water after 36 h.

### Freshwater crabs could accumulate AIV from surrounding water

Groups of 3 crabs were inoculated by incubating in viral water. As shown in Table 1, AIV could be detected in crabs’ gills and intestinal tracts since 1 hpi. The crabs’ gills accumulated AIV more efficiently than their gastrointestinal tracts. After 8 h of virus inoculation, the virus titer in crab gills was higher than that in water. At 36 hpi, the viral titers in crabs began to drop. Whereas, viral titer in water kept on dropping from the beginning of the experiment. AIV could still be detected in 1 crab’s gill at 36 hpi and in 1 crab’s gill and gastrointestinal tract at 48 hpi. No virus was detected in other organs of the crabs. It explained that AIV could “infect” crabs through their respiratory and digestive systems. There’s no detectable AIV after 48 hpi in neither crabs nor in water.

**Table 1:** AIV accumulation in freshwater crabs

Hours post inoculation (hpi)	Viral titer (log_10_TCID_50_/mL)^a^				
	Gill	Gastrointestinal tract	Hepatopancreas	Muscle	Water
0	–	–	–	–	4.25
1	2.62/2.02/2.01	2.82/2.62/–	–	–	3.75
3	3.14/3.07/2.83	3.11/–/–	–	–	3.50
8	4.96/4.91/4.61	2.92/–/–	–	–	2.75
12	4.88/4.45/4.18	2.18/–/–	–	–	3.25
24	4.97/3.94/–	–	–	–	2.50
36	2.68/–/–	–	–	–	2.25
48	2.70/–/–	2.62/–/–	–	–	1.50
60	–	–	–	–	–

^a^Five groups crabs (3 per group) were distributed into 5 viral water tanks. After 0, 1, 3, 8 and 12 h incubation, 1 group of 3 crabs were rinsed and euthanized. Crabs gills, gastrointestinal tract, hepatopancreas, muscle tissue and viral water were collected for viral titration in MDCK cells

–, no virus was detected in samples

### Freshwater crabs could accumulate AIV virus constantly

**Fig. 2:**
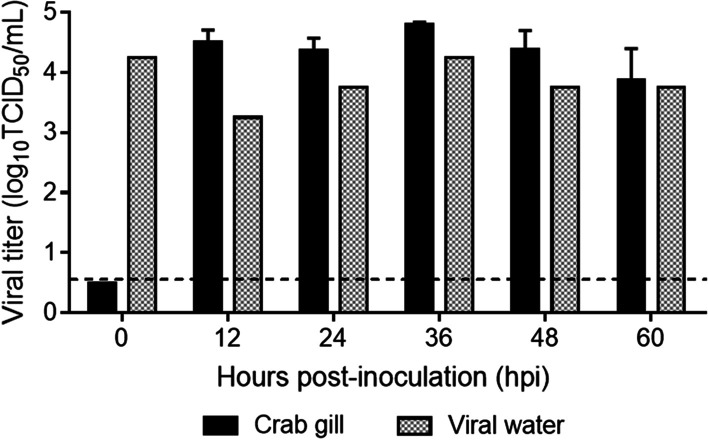
AIV accumulating limitation of crabs. Six groups of 3 crabs were transferred into 1 L viral water. Every 12 h, the tank water was changed with fresh viral water. One group of crabs were rinsed and euthanized for gills collection at designated time points. The viral water sample was collected at the same time. Crabs and water samples were titrated in MDCK cells. The data shown are the means of three replicates; the error bars indicate standard deviations. The dashed lines indicate the lower limit of virus detection

The crabs surrounding viral water was refreshed every 12 h. The viral titers in crabs were consistent but higher than viral water (As shown in Fig. 2). So the Chinese mitten crabs could continue accumulating AIV but might be confined by their size or viral titer in water.

### Freshwater crabs spread AIV to surrounding water and naïve crabs

We inoculated crabs by incubating them in viral water for 8 h and used them as the inoculated groups. Groups of 3 naïve crabs were treated as sentinel groups. As shown in Table 2, AIV could be detected from inoculated crabs until 8 hpi. Since 1 hpi, AIV was detected from 2 of the 3 sentinel crabs. At 2.5 hpi, AIV was only detected from 1 sentinel crab. There’s no detectable AIV in sentinel groups after 3 hpi. AIV was detected from water at 1 and 1.5 hpi. However, there was no live virus in the water after two hours. The inoculated groups contained higher viral titer than sentinel groups and water. The result indicated that, AIV contaminated crabs could spread virus into water and “infect” the naïve crabs.

**Table 2:** AIV spreading activity of freshwater crabs

Hours post co-cultured (hpc)	Viral titer (log_10_TCID_50_/mL)^a^		
	Inoculated crabs	Sentinel crabs	Water
0	4.96/4.91/4.61	–	–
0.5	4.05/3.71/3.67	–	–
1	3.74/3.62/3.18	2.89/2.34/–	2.50
1.5	3.84/2.85/2.35	2.95/2.45/–	2.25
2	3.68/3.17/2.13	2.60/2.35/–	–
2.5	3.24/2.68/2.16	2.68/–/–	–
3	2.49/2.32/–	–	–
3.5	2.70/–/–	–	–
4	2.57/–/–	–	–
8	2.49/–/–	–	–
12 to 60*	–	–	–

^a^Groups of 3 crabs were inoculated by incubating in viral water for 8 h. After rinsing thoroughly, the inoculated crabs were transferred into fresh water tanks. Groups of 3 sentinel crabs were put into each tank. At designated time points, 1 group of inoculated and sentinel crabs were rinsed and euthanized. Their gills and water were collected at the same time for viral titration in MDCK cells.

–, no virus was detected in samples

*Crabs and water samples were collected every 12 h

## Discussion

Pathogenic ecology of AIV including host, pathogen and environment. The biotic and abiotic factors of environment play important roles in AIV spreading process. Waterfowls especially the migratory waterfowls can disseminate AIV along their flyways. There’s high possibility that freshwater crabs could be contaminated by AIV shedding from the waterfowls.

Crabs could be good indicators reflecting the contaminating levels in their living environments. Crabs resided in the surfical sediment and might feed on contaminated sediments, so the aquatic contaminants surveillance conducting on crab could be more sensitive than on fish and water. It could be used as indicators for contaminants such as heavy metals, polychlorinated dibenzo-p-dioxins and dioxin-like (dl) PCBs [10, 11]. Researchers found AIV RNA in small aquatic vertebrates and invertebrates, such as water fleas [12], bamboo shrimp (*Atyopsis moluccensis*), clams (*Corbicula fluminea*), freshwater snails (*Physa spp.*), zebra mussels (*Dreissena ploymorpha*), crayfish and Mediterranean cone shell (Conus sp.). Experiments indicated these aquatic animals can accumulate AIVs through water filtering but the infectivity of these accumulated AIV had not been evaluated yet [13–18]. Our study for the first time evaluated the infectivity of accumulated AIV in crabs and determined the AIV spreading activity of freshwater crabs. The results indicated that freshwater crab could also act as an AIV indicator in aquatic environment. Viral contaminating levels in crabs might be included in the future AIV field surveillance activities.

Further study should be conducted to evaluate the AIV transmission between freshwater crabs and their predators.

## Conclusion

In the present study, we evaluated the accumulation and spreading of AIV in freshwater crabs. AIV could be accumulated in multiple organs of the crabs and stay infectious longer than in water. Most importantly, AIV could be carried by the crabs into freshwater and transmitted to the naïve crabs. Our study indicates that freshwater crabs are important factors in the AIV ecosystem.

## Data Availability

All data generated or analysed during this study are included in this published article.

## Abbreviations

AIV: Avian influenza virus
HA: Hemagglutinin
NA: Neuraminidase
MDCK: Madin-Darby canine kidney
TCID_50_: 50% Tissue culture infectious dose
h: Hours
hpi: Hours post inoculation
hpc: Hours post co-cultured
